# Phenylthiourea Specifically Reduces Zebrafish Eye Size

**DOI:** 10.1371/journal.pone.0040132

**Published:** 2012-06-27

**Authors:** Zeran Li, Devon Ptak, Liyun Zhang, Elwood K. Walls, Wenxuan Zhong, Yuk Fai Leung

**Affiliations:** 1 Department of Biological Sciences, Purdue University, West Lafayette, Indiana, United States of America; 2 Division of Biology & Biomedical Sciences, Washington University in St. Louis, St. Louis, Missouri, United States of America; 3 Department of Cellular and Integrative Physiology, Indiana University School of Medicine Lafayette, West Lafayette, Indiana, United States of America; 4 Department of Statistics, University of Illinois at Urbana-Champaign, Champaign, Illinois, United States of America; 5 Department of Biochemistry and Molecular Biology, Indiana University School of Medicine Lafayette, West Lafayette, Indiana, United States of America; Wayne State University School of Medicine, United States of America

## Abstract

Phenylthiourea (PTU) is commonly used for inhibiting melanization of zebrafish embryos. In this study, the standard treatment with 0.2 mM PTU was demonstrated to specifically reduce eye size in larval fish starting at three days post-fertilization. This effect is likely the result of a reduction in retinal and lens size of PTU-treated eyes and is not related to melanization inhibition. This is because the eye size of *tyr,* a genetic mutant of tyrosinase whose activity is inhibited in PTU treatment, was not reduced. As PTU contains a thiocarbamide group which is presented in many goitrogens, suppressing thyroid hormone production is a possible mechanism by which PTU treatment may reduce eye size. Despite the fact that thyroxine level was found to be reduced in PTU-treated larvae, thyroid hormone supplements did not rescue the eye size reduction. Instead, treating embryos with six goitrogens, including inhibitors of thyroid peroxidase (TPO) and sodium-iodide symporter (NIS), suggested an alternative possibility. Specifically, three TPO inhibitors, including those that do not possess thiocarbamide, specifically reduced eye size; whereas none of the NIS inhibitors could elicit this effect. These observations indicate that TPO inhibition rather than a general suppression of thyroid hormone synthesis is likely the underlying cause of PTU-induced eye size reduction. Furthermore, the tissue-specific effect of PTU treatment might be mediated by an eye-specific TPO expression. Compared with treatment with other tyrosinase inhibitors or bleaching to remove melanization, PTU treatment remains the most effective approach. Thus, one should use caution when interpreting results that are obtained from PTU-treated embryos.

## Introduction

Phenylthiourea (PTU) has long been demonstrated to inhibit melanization, the formation of black pigment, in various animals. Inhibition of melanization by PTU has been observed in rats [1], [2], ascidians [3], [4], [5], planarians [6], moths [7], frogs [8], [9], [10], salamanders [11], tropical fish [12] and zebrafish [13]. Specifically, PTU inhibits tyrosinase, a key enzyme in the melanogenic pathway [14], by binding to copper ions and by interacting with the side chains in the active site [15]. This pigment inhibition effect of PTU is reversible; once PTU is removed from the system, melanization will resume [1], [5].

In recent years, zebrafish (*Danio rerio*) has become a popular research model [16]. The relative transparency of zebrafish embryos compared with the other vertebrate models and its *ex vivo* mode of development can facilitate the visualization and imaging of the developmental process. In particular, melanization of zebrafish embryos can be conveniently inhibited by treating embryos with 0.003% (w/v) or 0.2 mM PTU (commonly referred to as 1X PTU) starting from about 12 hours postfertilization (hpf) to shortly before 24 hpf [17], [18], [19], [20], [21], [22]. It has also been reported that treating embryos with 0.075 mM or 0.375X PTU at about 24 hpf can inhibit melanization [23]. However, this approach is not very effective and embryos always contain considerable melanization after treatment [24]. Another way of suppressing melanization is by utilizing genetic mutants of pigmentation [25], including *sandy*/*tyr* [21], *nacre* [26] and *casper* (double mutant of *nacre* and *roy*, an iridophore mutant) [27]. Nonetheless, using mutant embryos is not as versatile as PTU treatment for many downstream investigations. Thus, PTU treatment remains the prime choice for inhibition of melanization.

Despite the convenience of PTU treatment in inhibiting melanization, it has been shown in several animal models that this treatment can produce undesirable side effects at the molecular and physiological levels. For example, it has been shown that PTU treatment perturbs gene expression in zebrafish embryos. These include an activation of *cytochrome P4501A1* (*cyp1a1*) [28] and a suppression of *retinol-binding protein 4* (*rbp4*) [29]. In ascidians, it has been demonstrated that treatment with 0.35 – 0.5 mM PTU slightly shortens the length and causes a curvature in the tails [5]. In addition, two other studies have reported that a lower concentration of PTU treatment at 0.001% prevents normal elongation of the notochord [3], and also causes short curved tails, immobility and a suppression of metamorphosis [3], [4]. In rats, it has been demonstrated that the administration of PTU through feeding and/or intraperitoneal injection can cause both acute and chronic toxicity [30]. Interestingly, the studies on rats have also led to the original discovery of thyroid hypertrophy and in turn the goitrogenic effect of PTU [31], [32], which has been subsequently confirmed in studies of the frog [8]. In zebrafish, it has been shown that treating zebrafish larvae with 0.003% PTU can suppress thyroid hormone (T4) immuno-reactivity in thyroid follicles [33]. A recent report by Bohnsack and colleagues showed that PTU treatment can alter retinoic acid and insulin-like growth factor regulation of neural crest and craniofacial development in zebrafish [34]. The authors also found that this PTU-induced problem is partially rescued by thyroid hormone supplement. However, it has also been demonstrated that the developmental abnormalities in ascidians caused by PTU treatment are not primarily due to anti-thyroid effect or pigment inhibition [3], [4]. Thus, PTU can potentially exert its effect on embryonic development through a variety of molecular mechanisms.

**Figure 1:**
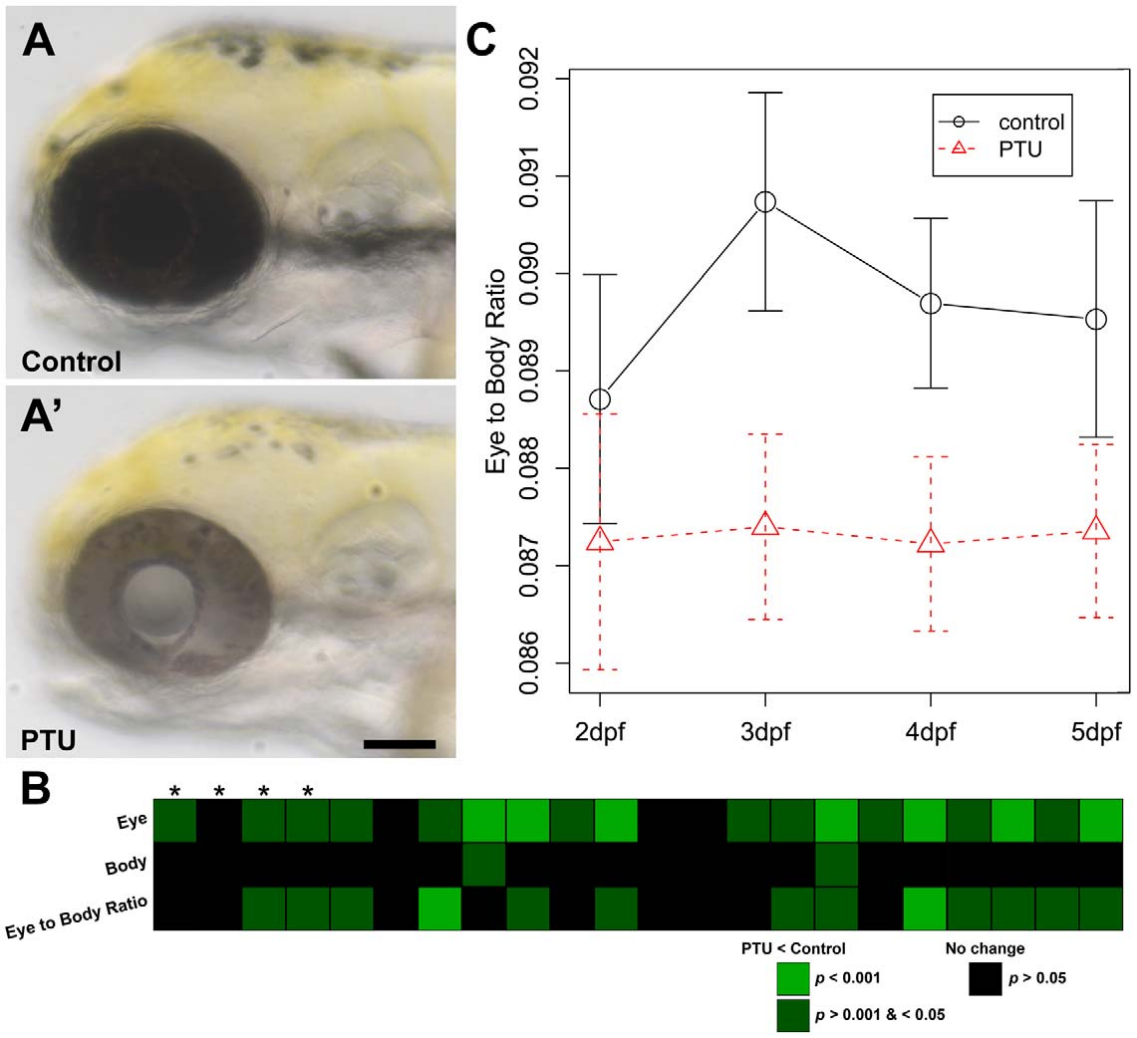
PTU treatment specifically reduces eye size starting at 3 dpf. (A & A') After PTU treatment (PTU), the production of black pigment or melanization in a larva was suppressed (A'). In addition, the eye size of the PTU-treated larvae also appeared smaller compared with the untreated siblings (control) (A) at 3 dpf. Scale bar  = 100 µm. (B & C) To quantify potential differences in eye size, the anterior-posterior length or area of eye and body were measured in multiple experiments conducted on different days. (B) A heatmap that shows the results from 22 independent experiments at 3 dpf. At least 10 embryos were used in each treatment group per experiment. Each column represents one experiment and the individual box represents the Holm-adjusted *p*-value of a Wilcoxon rank sum test for the comparison of eye or body measurements, or their ratios (eye/body size ratios) between PTU-treated and control larvae. The color of each box represents the difference in the means of the parameter between the two groups and the intensity represents the corresponding *p-*value of each test (green: PTU < control, *p-*value <0.001; dark green: PTU < control, *p*-value >0.001 & <0.05; black: PTU  =  control, *p-*value >0.05). The experiments analyzed by area measurements are highlighted by asterisks; otherwise, they were analyzed by length measurements. The PTU treatment specifically reduced the measurements of the eye size compared with the body size. See text for further supporting statistical analyses. (C) A plot of the means of eye/body length ratios of 15 control and PTU-treated larvae at 2–5 dpf obtained from one of the multi-days experiments. The error bars show the 95% confidence intervals.

In our own studies, we have noticed a possible reduction of eye size of zebrafish larvae after treatment with 1X PTU (Figure 1A). Several factors have been tested as the potential cause of this phenotype, including the batch and supplier of PTU, the starting time of PTU treatment (12 vs. 24 hpf), the specific experimenter (at least four researchers prepared the chemicals and treated embryos independently over the course of the project) and laboratory locations (the experiments have been conducted at two different locations). None of these factors appear to be associated with the eye size reduction, suggesting that it may be a specific effect of PTU treatment. In fact, such an effect on eye size reduction has been documented in frogs [8], [35], but has not been further studied. The current study describes the characterization of the effect of PTU treatment on eye development in zebrafish. In particular, the results provide experimental evidence that the PTU-induced effect on eye size reduction is not caused by a general inhibition of thyroid hormone production or melanization, but is likely mediated through a more specific inhibition of a thyroid peroxidase (TPO) or TPO-like activity in zebrafish embryos.

## Results

### PTU treatment specifically reduces eye size in zebrafish

To quantify the specific reduction of eye size, eye and body size of larvae treated with 1X PTU (PTU) and untreated siblings (control) were measured. Two types of parameters were used for measuring size, including anterior-posterior length and area. For each type of parameter, a ratio between the eye and body measurements was calculated (eye/body size ratio), which is essentially a normalized eye measurement. This ratio was used in subsequent analyses for eye-specific size change. Twenty-two independent experiments were conducted at three days postfertilization (dpf), with at least 10 embryos in each treatment group per experiment (total N  = 343 for each group). Length and area measurements were used in 18 and 4 experiments respectively. To compare the eye and body measurements and the eye/body size ratios between the control and PTU-treated groups in each experiment, Wilcoxon rank sum tests were conducted and the resulting *p-*values of the same type of measurements were adjusted for multiple hypothesis testing. The adjusted *p-*values are presented in a heatmap, in which the color of an individual box also represents the nature of the difference (Figure 1B; green: PTU < Control, *p-*value <0.001; dark green: PTU < Control, *p*-value >0.001 & <0.05; black: no change, *p-*value >0.05). Several patterns have been observed: first, there were more experiments (18/22 (81.8%)) in which the eye measurements were reduced by PTU treatment (*p*<0.05), compared with only 2/22 (9.1%) of the experiments in which the body measurements were reduced; second, the two experiments that had a reduction in body measurements had a concomitant reduction in eye measurements (*p*<0.05), but not vice versa. The *p*-value of the eye measurement reduction is at least equal to if not smaller than the body measurement reduction in both cases; third, there was a specific reduction of eye measurements relative to body measurements in 13/22 (59.1%) of the experiments, as determined by the eye/body size ratio. To evaluate the extent of this specific reduction, measurements from these 22 experiments were combined and analyzed by linear mixed-effects model. The effect on eye/body size ratio caused by PTU treatment was modeled as a fixed effect, while the experiments done on different days were modeled as a random effect. A separate model was fitted for the length and area measurements respectively. The analyses show that PTU treatment had a specific effect on the eye/body size ratio, regardless of the measurement type (length measurements: *F*(1, 521)  = 148.67, area measurements: *F*(1, 141)  = 24.97, *p-*value <0.001 in both cases). Specifically, the eye/body size ratios of the PTU-treated larvae were smaller than the control larvae (length measurements: 97.25% of controls, standard error (SE)  = 2.10%; area measurements: 96.52%, SE  = 2.05%; *p-*values  = 2.93e–30 and 1.70e–06 respectively). Together, these data indicate that PTU treatment preferentially reduces eye measurements compared with the whole body measurements. Length measurements were used in subsequent analysis unless specified otherwise.

To further quantify the effect of PTU treatment on eye size during embryogenesis, the eye and body length measurements of PTU-treated and control larvae (N = 15 for each group) were collected starting from 2 dpf for several days. Ten independent experiments were conducted and the results of one of these developmental series are shown in Figure 1C. By 3 dpf, the eye/body size ratios of the PTU-treated larvae were already measurably smaller (Wilcoxon rank sum test, Holm-adjusted *p*-value <0.05) in 6 out of 9 or 66.67% of the experiments that were conducted at 3 dpf. This is similar to the 59.1% frequency of occurrence as observed in the 3 dpf measurements as described in Figure 1B above. By 4 dpf, the eye/body size ratios of the PTU-treated larvae in 6 out of 9 or 66.67% of the measured experiments were smaller (*p*-value <0.05). While at 5 dpf, 3 out of 6 or 50% of the measured experiments still showed a size difference (*p*-value <0.05). Measurement of larvae older than 5 dpf was not conducted because this is the approximate stage when feeding usually begins [36]; and the behavioral variation in food consumption can be a confounding factor for morphological measurement. Nonetheless, a smaller eye/body size ratio in PTU-treated larvae between 3–5 dpf indicates that the observed reduction in eye size is not likely to be an overall developmental delay but rather a specific effect on eye development imposed by PTU treatment.

### PTU treatment reduces retinal and lens size but not retinal cell number

**Figure 2:**
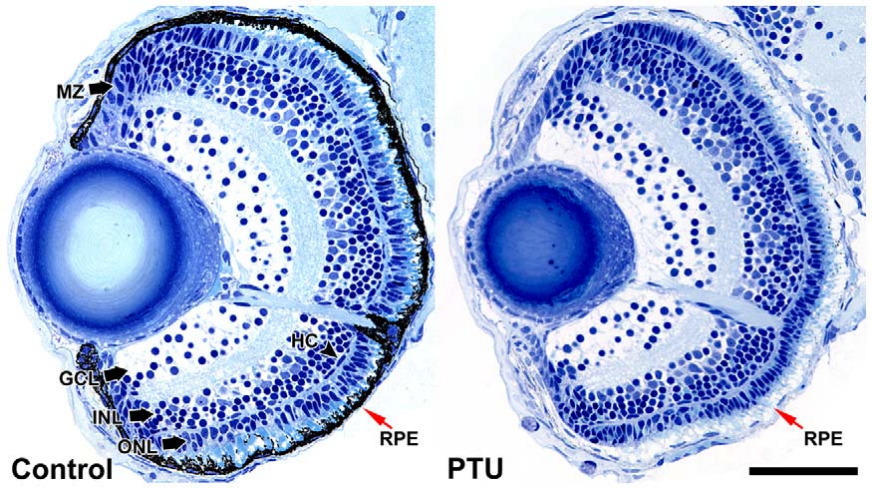
Histological analysis of zebrafish eyes before and after PTU treatment. Histological analysis was conducted on 1-μm-thick plastic sections of PTU-treated and control larvae at 4 dpf. The number of different recognizable cell types and categories was counted and the area of the retinas measured. Five sections were analyzed per condition. There are no differences in the counts for all cell types and categories, but the dorsal retinal and lens areas are smaller while the whole retinal area and cell density are marginally higher in the PTU-treated eyes. MZ: marginal zone, HC: horizontal cells, GCL: ganglion cell layer, INL: inner nuclear layer, ONL: outer nuclear layer. The RPE is indicated by red arrows. Scale bar  = 50 µm.

To investigate whether PTU treatment would alter retinal cell number, five plastic sections of PTU-treated and control larvae were collected and analyzed at 4 dpf (Figure 2). The number of cells in dorsal and ventral retina, defined as cells found dorsal or ventral to the optic nerve, was counted from both eyes. The categories that were counted include GCs, HCs, photoreceptors, cells in the proliferative marginal zone and cells in the inner nuclear layer with and without HCs. The retinal and lens areas were also measured from these samples. A linear mixed-effects model was used to compare the corresponding measurements between PTU-treated and control larvae with individual larva modeled as a random effect. There were no differences in any cell counts between these two groups (*p*-value >0.05 in all cases). However, the dorsal retinal areas were smaller in the PTU-treated group (mean 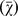 = 11870 µm^2^, standard deviation (*s*) = 564 µm^2^) compared with the control group (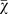 *= *13560 µm^2^, *s* = 1138 µm^2^) (*F*(1, 8)  = 8.83, *p*-value  = 0.018); while the ventral retinal areas were not different between the two groups (PTU-treated: 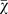 *= *6701 µm^2^, *s* = 530 µm^2^; control: 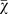 = 7103 µm^2^, *s* = 751 µm^2^; *F*(1, 8)  = 0.96, *p*-value  = 0.36). This resulted in the whole PTU-treated retinas being marginally smaller than the controls (PTU-treated: 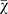 *= *18570 µm^2^, *s* = 1066 µm^2^; control: 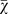 = 20660 µm^2^, *s* = 1853 µm^2^; *F*(1, 8)  = 4.78, *p*-value  = 0.06). Further, the lens were also smaller in the PTU-treated group (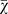 *= *5729 µm^2^, *s* = 968 µm^2^) compared with the control group (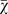 *= *7081 µm^2^, *s* = 593 µm^2^) (*F*(1, 8)  = 6.85, *p*-value  = 0.031). The cell density (cell count/retinal area) of the PTU-treated larvae was marginally higher than the controls on the dorsal (PTU-treated: 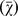 *= *0.037 µm^2^, *s* = 0.0011 µm^2^; control: 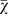 = 0.034 µm^2^, *s* = 0.0024 µm^2^; *F*(1, 8)  = 4.38, *p*-value  = 0.070) and whole retina (PTU-treated: 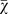 *= *0.036 µm^2^, *s* = 0.0014 µm^2^; control: 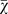 = 0.034 µm^2^, *s* = 0.0022 µm^2^; *F*(1, 8)  = 4.39, *p*-value  = 0.069). Additionally, cell density of ganglion cell layer (GCL), inner nuclear layer (INL), outer nuclear layer (ONL), and marginal zone were individually analyzed on dorsal, ventral and whole retina, but none of them showed any differences between PTU-treated and control larvae (*p*-values >0.05). Together with the morphometric measurements, the results indicate that the reduction in eye size caused by PTU treatment is likely a result of a general reduction of dorsal retinal and lens size.

### PTU treatment does not alter differentiation, proliferation and apoptosis of the retina

**Figure 3:**
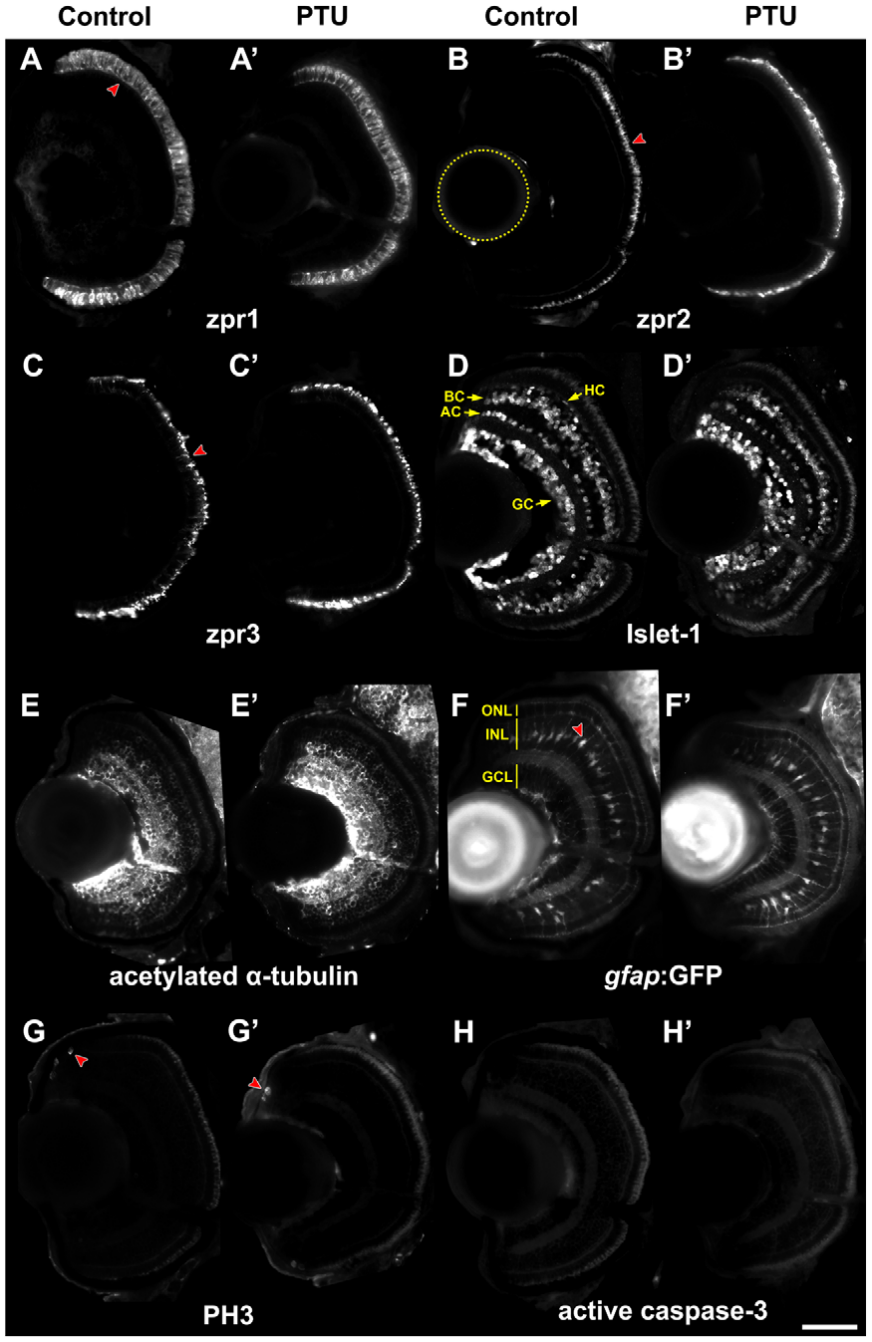
Immunohistochemical analysis of zebrafish eyes before and after PTU treatment. Immunostaining was conducted on 10-μm-thick cryosections of PTU-treated and control larvae at 4 dpf with the following first antibodies: (A & A') anti-zpr1 for cones (red arrowhead), (B & B') anti-zpr2 for RPE (red arrowhead), (C & C') anti-zpr3 for rods (red arrowhead), (D & D') anti-Islet1 for GCs, ACs, BCs and HCs, (E & E') anti-acetylated α-tubulin for the axons of differentiated neurons, (G & G') anti-PH3 for mitotic cells (red arrowheads) and (H & H') anti-active caspase3 for apoptotic cells. (F & F') MCs were visualized by cryosectioning of a transgenic fish *Tg(gfap:GFP)^mi2001^* treated with PTU in the same manner. The cell bodies were visualized by green fluorescence (red arrowhead). All positive signal areas or cell counts were extracted and normalized by retinal area, which was traced in the channel with DAPI nuclei stain. There are no differences in staining of any markers between the PTU-treated and control groups except for zpr2, in which the positive signal/ retinal area was larger in the PTU-treated group. All images are transverse sections; the lens is on the left (the location is indicated by a dotted yellow circle in (B)) and dorsal is up. The approximate retinal cell layers and cell types are indicated in (D) and (F). Scale bar  = 50 µm.

To further assess potential histological changes in the PTU-treated eyes, immunostaining experiments were conducted on cryosections at 4 dpf with immunohistochemical markers for major cell types in retina, retinal pigment epithelium (RPE), apoptosis and proliferation (Figure 3; N  =  at least 5 in each condition). The positive signal area or cell count was normalized to the corresponding retinal area for comparisons. First, there were no changes in the normalized positive-signal area or the overall pattern of the staining between the PTU-treated and control larvae. This was true for all major retinal cell types, including cones (anti-zpr1; Figure 3A & A'; Wilcoxon rank sum test, *p-*value  = 0.22), rods (anti-zpr3; Figure 3C & C'; Wilcoxon rank sum test, *p-*value  = 0.84), ganglion cells (GCs), amacrine cells (ACs), bipolar cells (BCs) and horizontal cells (HCs) (anti-Islet-1; Figure 3D & D'; Wilcoxon rank sum test, *p-*value  = 0.13). To analyze Müller cells (MCs), fluorescence signals in the retinas of *Tg(gfap:GFP)^mi2001^* transgenic larvae were compared [37]. Again, there was no difference between the number of GFP+ cells per retinal area between PTU-treated and control larvae (Figure 3F & F') (Wilcoxon rank sum test, *p*-value  = 0.34). These results suggest that PTU treatment does not affect the differentiation of major retinal cell types. Indeed, the failure of PTU treatment to influence cellular differentiation in the retina was also confirmed by the lack of a difference in the immunostaining result of the axons of differentiated neurons (anti-acetylated α-tubulin; Figure 3E & E') (Wilcoxon rank sum test, *p*-value  = 0.42). Second, the only difference between PTU-treated larvae compared with the controls was an increase in the zpr2+ RPE signal per retinal area (Figure 3B & B'; Wilcoxon rank sum test, *p*-value  = 0.0079). This result is probably due to the formation of melanophores in the wild-type (WT) RPE cells, which might have interfered with the fluorescence emission. Third, there was no apparent change in the proliferation status, as indicated by a comparable number of mitotic cells between PTU-treated and control larvae (anti-PH3; Figure 3G & G') (Wilcoxon rank sum test, *p*-value  = 0.29). The analyses of this marker at earlier developmental stages including 36, 48 and 60 hpf also did not show any difference (*p*-values  = 0.71, 0.34 and 0.9 respectively). Fourth, the observation that there was rarely any anti-active caspase3 staining in the retinas (Figure 3H & H') strongly suggests that PTU-treatment did not induce apoptosis in the retinas. Thus, these experiments indicate that PTU treatment did not alter differentiation, proliferation, apoptosis or cell numbers of the retina. The effect of PTU treatment on RPE staining was probably due to the inhibition of melanization.

### PTU's effect on eye size reduction is not caused by inhibition of melanization

Since PTU treatment suppresses melanization by inhibiting tyrosinase, it is logical to hypothesize that the PTU effect on eye size reduction may be mediated through alteration of the pigmentation pathway. To test this hypothesis, eye and body lengths of *tyr^tk20/tk20^*, a genetic mutant of tyrosinase [25], were measured. *Tyr^tk20/tk20^* mutants cannot form any black pigment and have a similar morphology compared with the PTU-treated embryos. Five independent experiments were conducted on *tyr^tk20/tk20^* and WT siblings with normal melanization, and a total of 70 larvae were measured in each condition at 3 dpf. The results from a linear fixed-effects model analysis indicate that the genotype of the larvae did not change the eye/body size ratio (*F*(1, 136)  = 2.03, *p-*value  = 0.16). Subsequent measurements in two experiments on older 4 dpf larvae (N = 20 for each condition) also showed that the genotype did not affect eye/body size ratio when compared with WT siblings at the same stage (*F*(1, 37)  = 2.03, *p-*value  = 0.09). Thus, the results indicate that altered activity of tyrosinase and the resulting change in the levels of downstream metabolites in the melanization pathway do not affect eye size. The eye size reduction caused by PTU treatment is likely due to an uncharacterized chemical property of PTU.

### PTU treatment inhibits thyroid hormone production but its specific effect on eye size reduction is not rescued by thyroid hormone supplement

**Figure 4:**
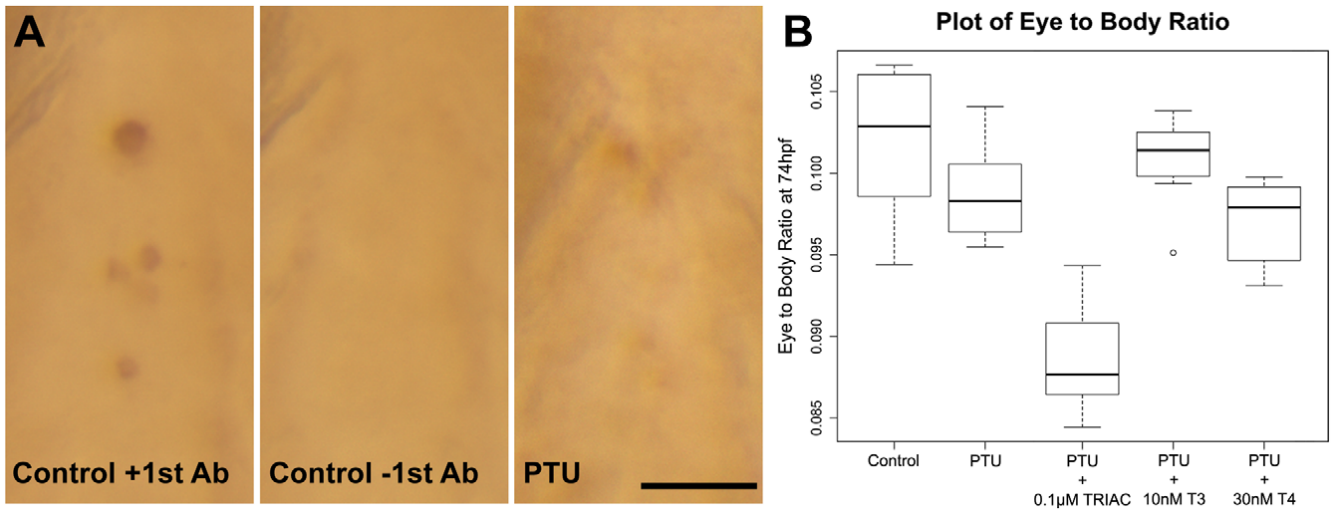
PTU suppresses thyroid hormone production but its specific inhibition on eye growth is not related to a general suppression of thyroid hormone synthesis. (A) Whole-mount immunostaining against T4 was performed on PTU-treated and control larvae at 4 dpf. In controls, the T4+ thyroid follicles were stained as dark brown spots along the ventral pharyngeal midline. This staining was substantially diminished or absent in the PTU-treated larvae and in the control without the first antibody. Scale bar  = 50 µm. (B) A boxplot of eye/body size ratios from an experiment of thyroid hormones supplements along with PTU treatment. Specifically, 10 larvae collected from the same parents were treated with nothing (control), PTU, PTU with 0.1 µM TriAc, 10 nM T3 or 30 nM T4. While the T3 supplement seemed to increase the eye/body size ratio of the PTU-treated larvae, statistical analysis on this and six additional independent experiments indicate that this was not a genuine increase. See text for details.

It has been reported that PTU treatment inhibits thyroid hormone production [33], which can be the underlying cause of eye size reduction. Indeed, whole-mount immunostaining against thyroxine (T4) carried out on PTU-treated and control larvae at 4 dpf shows that the staining of T4+ thyroid follicles was substantially reduced or absent in the PTU-treated group (Figure 4A). Therefore, these observations suggest that reductions in thyroid hormone levels by PTU treatment might play a role in eye size reduction. To test this hypothesis, different thyroid hormones were respectively added into the E3 medium with PTU to rescue the eye size of PTU-treated larvae. These include 30 nM T4, 10 nM triiodothyronine (T3), and 0.1 µM 3,3′,5-triiodothyroacetic acid (TriAc) [38], a thyroid hormone analogue.

The effectiveness of these three thyroid hormones was determined by quantitative PCR (qPCR) of two reporter genes, *thyroid hormone receptor α (TRα)* and *type II iodothyronine deiodinase (D2).* The expression of these two genes in zebrafish larvae has been shown to increase and decrease respectively after treatment with exogenous thyroid hormones [39]. The total RNAs for this qPCR analysis were extracted from the larvae whose eye and body lengths were measured (Figure 4B) in order to correlate any morphological changes with the altered thyroid hormone activity. Three technical replications were conducted. Compared with the untreated controls, all three thyroid hormones could induce an increase and a decrease in the expression of *TRα* and *D2* respectively as expected (PTU+TriAc vs. control – *TRα*: (fold change [range of fold change]) 10.84 [8.25–14.25], *D2*: 0.28 [0.20–0.40]; PTU+T3 vs. control – *TRα*: 2.33 [1.78–3.06], *D2*: 0.77 [0.58–1.03]; PTU+T4 vs. control – *TRα*: 6.12 [4.65–8.05], *D2*: 0.45 [0.25–0.78]). These results suggest that the thyroid hormones used in the rescue experiments were effective. It should be noted that the range of the fold change of *D2* in the PTU+T3 group overlaps with one; nonetheless, its positive effect on the expression level of *TRα* still supports that the T3 treatment was effective.

To determine the effect on eye growth by these combined thyroid hormone and PTU treatments, eye and body lengths were measured and their ratio calculated from 10 larvae in five treatment groups at 105 hpf. These larvae were treated with nothing (control), with PTU only, and with PTU plus TriAc, T3 or T4 as described above (Figure 4B). All embryos were collected from the same parents to ensure comparability. An ANOVA indicates that the chemical treatments had an effect on eye/body size ratio (*F*(4, 45)  = 41.05, *p-*value  = 1.8e–14). Specifically, the eye/body size ratios of the PTU-treated larvae were smaller compared with the controls (Bonferroni-adjusted *p-*value  = 0.048), while the ratios of PTU+T4 and PTU+T3 treatment groups were not different from the PTU-treated group (Bonferroni-adjusted *p-*values  = 0.77 & 0.33 respectively). An unexpected finding was that the eye/body size ratio of the PTU+TriAc treatment group was smaller than the PTU-treated group (Bonferroni-adjusted *p-*value  = 1.09e–10).

Despite an insignificant *p-*value in the statistic comparison, the PTU+T3 group was the only group that apparently had a larger median eye/body size ratio compared with the PTU-treated group (Figure 4B). To determine whether this was an artifact in the experiment or a potential rescue effect by T3, another six independent experiments were conducted at 3 dpf (The total N for the control, PTU-treated and PTU+T3 treatment groups in all experiments was 65). The results from a linear mixed-effects model indicate that the treatments altered the eye/body size ratio (*F*(2, 188)  = 9.03, *p-*value  = 2e–04). However, *post hoc* comparisons indicate that there was no difference in the ratio between PTU-treated and PTU+T3 treatment groups (Bonferroni-adjusted *p-*value  = 0.55); and only the PTU-treated group had a reduced eye/body size ratio compared with the control group (Bonferroni-adjusted *p-*value  = 0.0059). The lack of a clear rescue effect by T3 was not likely caused by an insufficient treatment time, as when two of these experiments were measured again on day 4, none showed a difference in the eye/body size ratio between the PTU+T3 treatment and PTU-treated groups (Bonferroni-adjusted *p-*value  = 0.64). Together, the data suggest that while PTU treatment suppresses thyroid hormone synthesis, this disruption in thyroid hormone production is not likely the cause of the specific reduction of eye size in the PTU-treated larvae.

### Only inhibitors of thyroid peroxidase, and not other types of goitrogens, specifically reduce eye size

PTU inhibits TPO, one of the two molecular targets in the thyroid hormone synthesis pathway that common goitrogens would target to [40]. TPO catalyzes the oxidation of iodide in the thyroid follicle and in turn facilitates the iodination of thyroglobulin [41]. The other molecular target of common goitrogens is sodium-iodide symporter (NIS) which mediates the first step in the thyroid hormone synthesis by transporting iodide ions into the thyroid follicular cells [42], [43].

To further rule out the possible relationship between the suppression of thyroid hormone synthesis and the specific reduction of eye size in development, six goitrogens, including 6-n-propyl-2-thiouracil (PTuracil) [33], methimazole (MM) [33], 2, 3-dihydroxypyridine (DHP) [40], [44], resorcinol (RES) [40], potassium perchlorate (KClO4) [33] and potassium thiocyanate (KSCN) [43] were used to treat developing embryos. These six chemicals belong to three general classes: 1) inhibitors of TPO with thiocarbamide (PTuracil & MM), the same functional group as in PTU, 2) inhibitors of TPO without thiocarbamide (DHP & RES), and 3) inhibitors of NIS (KSCN and KClO4). Specifically, 0.025% PTuracil, 4 mM MM, 1 mM DHP, 0.5 mM RES, 0.2% KClO4 and 10 mM KSCN were added into the E3 medium as in the PTU treatment respectively. Multiple independent replications were conducted and at least 10 larvae were used in each of the chemical treatment and control group in each repeat.

**Figure 5:**
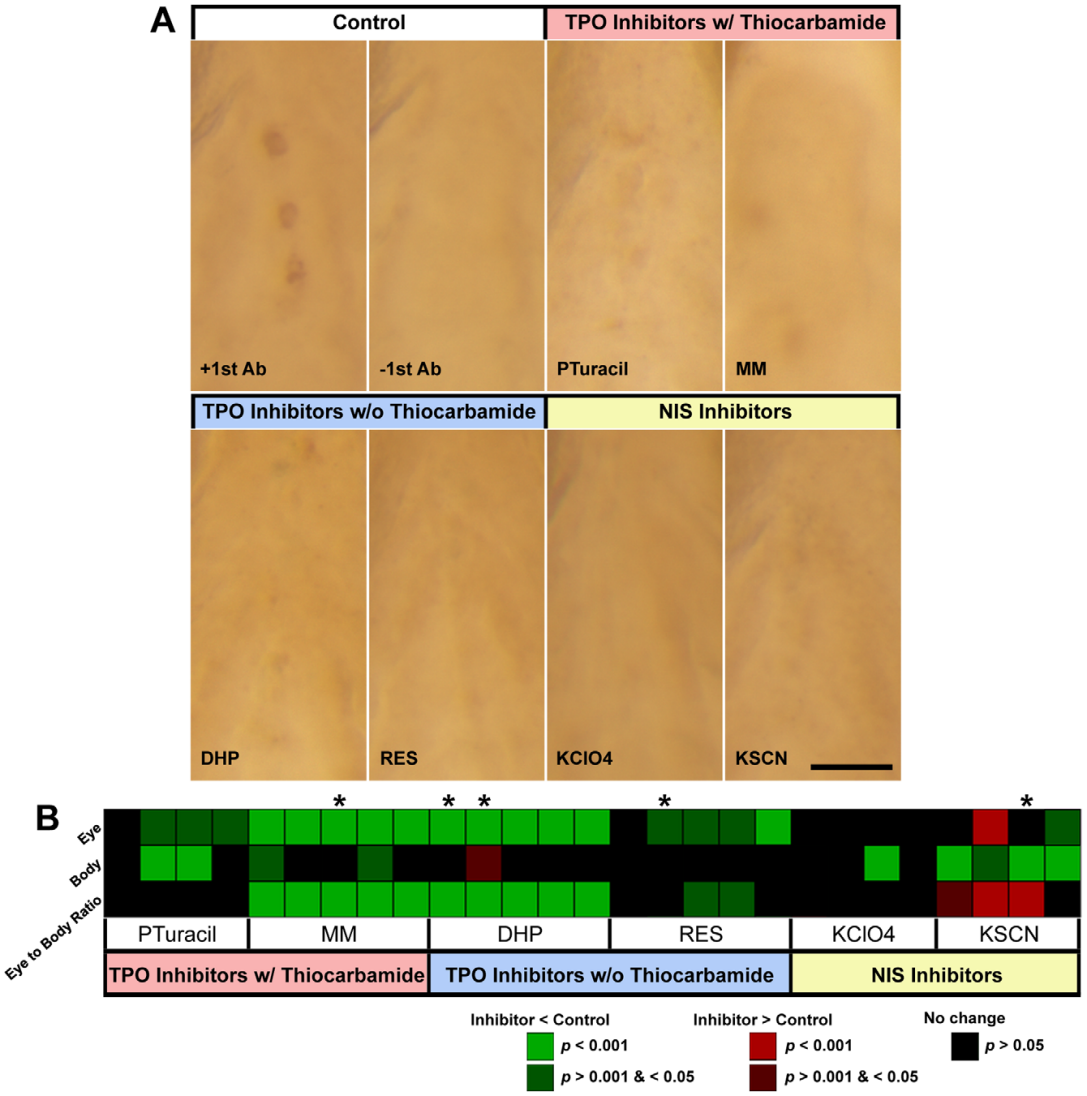
The suppression of thyroid hormone synthesis by six goitrogens reveals that PTU suppression on eye growth is related to inhibition of TPO. (A) The six goitrogens used include two TPO inhibitors with thiocarbamide group: 0.025% PTuracil and 4 mM MM; two TPO inhibitors without thiocarbamide group: 1mM DHP and 0.5mM RES; and two NIS inhibitors: 0.2% KClO4 and 10mM KSCN. All these chemicals were used to treat zebrafish embryos in the same way as PTU. Whole-mount immunostaining against T4 was performed at 4 dpf as in Figure 4. In controls, T4+ thyroid follicles were stained as dark brown spots along the ventral pharyngeal midline. This staining was substantially diminished or absent in the inhibitors-treated groups and in the control without the first antibody. Scale bar  = 50 µm. (B) A heatmap that shows the results from multiple experiments in which the larvae were independently treated with these six goitrogens. These include two TPO inhibitors with thiocarbamide group: 0.025% PTuracil (N of independent experiments  = 4) and 4 mM MM (N = 5); two TPO inhibitors without thiocarbamide group: 1mM DHP (N = 4), 0.5mM RES (N = 5); and two NIS inhibitors: 0.2% KClO4 (N = 4) and 10mM KSCN (N = 4). All these chemicals were used to treat zebrafish embryos the same way as PTU and at least 10 larvae were used in each individual treatment in each replication. The eye and body lengths were measured and the corresponding eye/body size ratios calculated. The eye and body measurements and their ratios between inhibitors-treated and control larvae were compared by Wilcoxon rank sum test, and the results were presented in a heatmap as in Figure 1. The color of each box represents the difference in the means of the parameter and the intensity represents the corresponding Holm-adjusted p-value of each test (green: inhibitor < control, p-value <0.001; dark green: inhibitor < control, p-value >0.001 & <0.05, black: inhibitor  =  control, p-value >0.05; dark red: inhibitor > control, p-value >0.001 & <0.05; red: inhibitor > control, p-value <0.001). The experiments analyzed at 4 dpf are highlighted by asterisks; otherwise they were analyzed at 3 dpf. A linear mixed-effects model was fitted to analyze the effect of individual inhibitors on the measured parameters at 3 dpf. In general, three (MM, DHP & RES) out of four TPO inhibitors specifically reduced eye/body size ratio while none of the NIS inhibitors did. These observations suggest that a general suppression of thyroid hormone synthesis is not the cause of the specific eye size reduction, but a specific inhibition of TPO or TPO-like activity can reduce eye size.

The effects of the treatment of these chemicals on thyroid hormone synthesis were determined by whole-mount immunostaining as described above (Figure 5A) and the eye and body lengths of the larvae measured at 3 or 4 dpf (Figure 5B). Each individual experiment was analyzed by Wilcoxon rank sum test and the Holm-adjusted *p-*values of the same type of treatment were presented in a heatmap (green: Inhibitor < Control, *p-*value <0.001; dark green: Inhibitor < Control, *p*-value >0.001 & <0.05; red: Inhibitor > Control, *p-*value <0.001; dark red: Inhibitor > Control, *p-*value >0.001 & <0.05; black: no change, *p-*value >0.05). In addition, all treatment repeats of the same chemical at 3 dpf were analyzed by fitting a linear mixed-effects model as described above. In general, while all chemicals were effective in reducing the staining of T4+ thyroid follicles (Figure 5A), the magnitude of treatment effects on eye size reduction varied. First, the eye/body size ratios of larvae treated with TPO inhibitors, except for PTuracil, were decreased compared with their control siblings (MM: 90.77% of control, SE  = 1.15%, *F*(1, 76)  = 172.21, *p-*value  = 3.20e–21; DHP: 89.26%, SE  = 1.70%, *F*(1, 56)  = 222.50, *p-*value  = 3.69e–21; RES: 97.4%, SE  = 2.08%, *F*(1, 75)  = 25.37, *p-*value  = 3.18e–06; PTuracil: 100.78%, SE  = 3.98%, *F*(1, 76)  = 3.11, *p-*value  = 0.082). Second, the eye reduction effect induced by TPO inhibitors without thiocarbamide was relatively specific. In fact, there was no reduction of body length in larvae treated by DHP (*F*(1, 56)  = 0.01, *p-*value  = 0.92) and RES (*F*(1, 75)  = 0.79, *p-*value  = 0.38). Third, the eye/body size ratios were generally larger in the larvae treated with the two NIS inhibitors, KClO4 (101.3% of control, SE  = 5.95%, *F*(1, 75)  = 6.58, *p-*value  = 0.012) and KSCN (103.68%, SE  = 0.99%, *F*(1, 56)  = 28.25, *p-*value  = 1.92e–06). This effect was actually caused by a reduction of body length (KClO4: *F*(1, 75)  = 24.09, *p-*value  = 5.21e–06; KSCN: *F*(1, 56)  = 41.05, *p-*value  = 3.29e–08) but not eye length (KClO4: *F*(1, 75)  = 1.43, *p-*value  = 0.24; KSCN: *F*(1, 56)  = 1.18, *p-*value  = 0.28).

While these chemicals are specific thyroid inhibitors, it is possible that they mediate their effects on growth by other non-specific changes, including osmolarity and pH, in the E3 medium. Thus, osmolarity and pH of these chemicals diluted in E3 were measured (Table 1). At least two measurements were conducted with independently prepared chemicals with different water sources, and three technical replicates were used in each measurement. For osmolarity measurements, an ANOVA shows that the addition of chemicals could change the osmolarity of the resulting solutions (*F*(7,50)  = 581.6, *p-*value <2.2e–16). The *post hoc* analysis indicates that the addition of KClO4, KSCN and MM increased the osmolarity compared with the control (Bonferroni-adjusted *p*-values  = 3.94e–42, 4.76e–30 and 8.36e–7 respectively) while the addition of the other chemicals did not (*p*-values >0.05) (Table 1). For pH measurements, a two-way ANOVA shows that there was a main effect of chemical treatments (*F*(7,32)  = 111.11, *p-*value <2.2e–16) and water sources (*F*(1,32)  = 137.51, *p-*value  = 4.01e–13). Specifically, the addition of KClO4 and KSCN decreased the pH of the solution (Bonferroni-adjusted *p*-values: 3.58e–8 and 6.96e–6) while the addition of the other chemicals did not (*p*-values >0.05) (Table 1). These results do not support the possibility that changes in osmolarity and pH would specifically decrease the eye size in zebrafish larvae because (1) KClO4 and KSCN solutions increased the eye/body size ratio and did not alter the absolute eye size, despite having the highest increase and decrease in osmolarity and pH respectively, (2) the addition of PTU did not alter the osmolarity or pH of the E3, and (3) TPO inhibitors do not generally change osmolarity and pH. The only exception was MM, which modestly increased the osmolarity of E3.

**Table 1:** Properties of the solutions used to treat developing zebrafish larvae.

Chemical	Percentage of chemical in E3 (%)	Molarity of chemical in E3 (mM)	Osmolarity of chemical in E3 ( 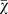 ±*s*) mOsm Kg^−1^	pH measurements; water source 1 ( 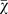 ±*s*)	pH measurements; water source 2 ( 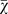 ±*s*)
Control (E3 only)	-	-	11.63±1.19	5.68±0.04	6.17±0.09
PTU + E3	0.003	0.2	11.75±1.58	5.68±0.03	5.94±0.02
PTuracil + E3	0.02	1.175	12.75±0.89	5.75±0.02	5.99±0.08
MM + E3	0.046	4	15.63±1.19	5.63±0.03	5.69±0.06
DHP + E3	0.011	1	12.00±0.63	5.66±0.01	5.57±0.02
RES + E3	0.0055	0.5	11.33±0.82	5.64±0.01	5.52±0.02
KClO_4_ + E3	0.2	14.435	42.00±2.27	5.43±0.03	5.78±0.01
KSCN + E3	0.0972	10	30.00±0.00	5.49±0.03	5.36±0.04

The osmolarity and pH of the chemicals in E3 were determined. For osmolarity, the measurements are combined from at least two different experiments because there was no difference between individual experiments (ANOVA, *F*(1,56)  = 0.24, *p-*value  = 0.62). See text for details. While for pH, the measurements of solutions prepared with two different sources of water were different (ANOVA, *F*(1,46)  = 5.35, *p*-value  = 0.025); thus each preparation is presented separately.

Since only inhibitors of TPO and not NIS can specifically reduce eye size in a way that is highly similar to that in the PTU-treated larvae, the data also argue against the role of a general suppression of thyroid activity in causing a specific eye size reduction. Furthermore, the absence of the thiocarbamide functional group in TPO inhibitors including RES and DHP, and their effectiveness in reducing eye size without affecting body size strongly argue for the role of Tpo in PTU-induced eye size reduction.

### Tpo is expressed in the mouse retina

**Figure 6:**
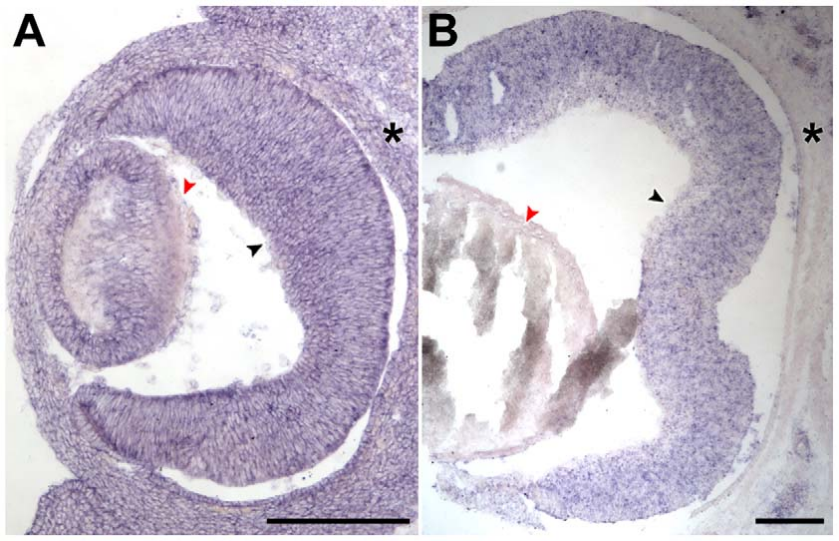
*Tpo* is specifically expressed in developing mouse retinas. *In situ* hybridization was conducted with a riboprobe specific for *Tpo* on 20-µm-thick cryosections obtained from mouse embryos at E13.5 (A; transverse section) and P0 (B; coronal section). (A) At E13.5, there was a general expression of *Tpo* in the whole embryo. (B) By P0, there was a specific expression of *Tpo* in the retina (black arrowhead) compared with lens (red arrowhead) and the surrounding periocular tissue (asterisks). Scale bar  = 200µm for both (A) and (B).

The specific effect of TPO inhibition on eye size suggests that this effect can be localized in the proximity of the eye rather than caused by a general suppression of thyroid hormone synthesis. This led to the hypothesis that there was a Tpo or Tpo-like activity in the eye region. Since the zebrafish *tpo* sequence is still partial and tentative (ZFIN ID: ZDB-GENE-110519-2), the expression of mouse *Tpo* was analyzed by *in situ* hybridization on cryosectioned mouse embryos at stages E13.5 and P0. These periods cover the developmental stages when the early-born cell types (GCs, HCs, ACs and cones) and all retinal cell types can be detected in the retina respectively [45]. The *in situ* results show that there was a strong general expression of *Tpo* in the mouse embryo including the retina and lens at E13.5 (Figure 6A). By P0, the expression of *Tpo* became localized in several regions in the embryo, including a relatively specific expression in the retina compared with the surrounding tissue (Figure 6B). The expression pattern of *Tpo* supports a possible localized role in growth regulation by eye-specific TPO.

### Other tyrosinase inhibitors do not inhibit pigmentation as well as PTU or will have adverse side effects

Since PTU has been widely used as an inhibitor of melanization for zebrafish research, the identification of its specific effects on eye size indicates the need for a better alternative. To search for alternative inhibitors for melanization, five tyrosinase inhibitors were tested. These include cetylpyridinium chloride (CPC) [46], 2-mercaptobenzothiazole (MBT) [47], mequinol (MQ) [48], 4-hydroxybenzyl alcohol (4HBA) [49] and 1-hydroxy-3-phenylurea (HPU) [50]. CPC and 4HBA are irreversible inhibitors of tyrosinase and are reported to be more potent than PTU [51], 10 µM MBT has been reported as an equally good inhibitor of melanization as PTU for zebrafish [47], MQ is a competitive inhibitor and is commonly used in prescription skin lightener [48], while HPU has been reported to be more potent than PTU [50]. A titration series of these chemicals except for MBT was tested, and the resulting effect on gross morphology and pigment inhibition observed. The specific concentration ranges tested for these chemicals are as follows: CPC: nine concentrations between 0.03 – 0.25 mM; MQ: nine concentrations between 0.075 – 1 mM; 4HBA: 15 concentrations between 0.3 – 0.9 mM; HPU: nine concentrations between 0.05 – 1.5 mM. All these concentration ranges cover the reported inhibitory concentrations, except for the range for MQ, which is at least 100 times lower than the standard formulation in the skin lightener (2% or ∼ 161 mM).

**Figure 7:**
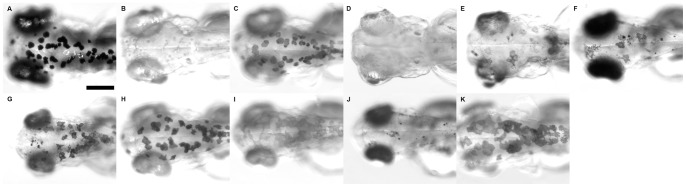
Morphology of zebrafish after treatment with various tyrosinase inhibitors. Different tyrosinase inhibitors, when applied at the same stage as PTU singly or in combination, suppress pigmentation to a different extent at 3 dpf. The inhibitors and their combinations include: (A) untreated control, (B) 1X PTU, (C) 0.3X PTU, (D) 10 µM MBT, (E) 0.25 mM MQ, (F) 0.7 mM 4HBA, (G) 0.8 mM HPU, (H) 0.3X PTU +0.7mM 4HBA, (I) 0.3X PTU +0.8 mM HPU, (J) 0.8 mM HPU +0.7 mM 4HBA and (K) 0.3X PTU +0.8 mM HPU +0.7 mM 4HBA. Scale bar  = 100 µm.

The effect of these chemicals on melanization varied. First, the embryos treated with all concentrations of CPC died within 24 hours of treatment. Second, the treatments of embryos with various concentrations of MQ (Figure 7E), 4HBA (Figure 7F) or HPU (Figure 7G) singly could not completely inhibit melanization, particularly at the concentrations that would show minimal abnormalities in development. Only 10 µM MBT (Figure 7D) could inhibit melanization as effectively as 1X PTU (Figure 7B) as previously reported. Nonetheless, the eye size of the MBT-treated larvae was substantially reduced compared with the controls at 3 dpf. In particular, the mean of eye/body size ratios of the treated larvae was 80.6% of untreated controls (Wilcoxon rank sum test, *p-*value  = 1.08e–5), indicating the eye was relatively smaller in the treated larvae (Figure 7D). Subsequently, a combination treatment with several chemicals at suboptimal concentrations including 0.3X PTU (Figure 7C), 0.7 mM HBA (Figure 7F) and 0.8 mM HPU (Figure 7G) was tested for any potential synergistic effects on melanization inhibition. However, none of the combinations tested could completely inhibit melanization either (Figure 7H–J). Furthermore, it was also noticed that two treatments with 0.7 mM 4HBA changed the morphology of the melanocytes (Figure 7F & J), indicating that this chemical may also affect melanocyte development in addition to melanization inhibition. Interestingly, when the same concentration of 4HBA was added together with 0.3X PTU, the effect on the melanocytes morphology diminished (Figure 7H & K). The treatments with HPU also showed a similar but weaker interaction effect with PTU as in the case of 4HBA (Figure 7G & I). One possible explanation is that PTU, 4HBA and HPU are competing for the same binding site in tyrosinase and PTU is the most effective in binding with the enzyme, thus in the combination treatment, PTU can effectively occupy the binding site of the enzyme and alleviate the toxic effects that may be elicited by the other chemicals. Nonetheless, these results indicate that PTU remains the most effective chemical inhibitor for zebrafish melanization with the least side-effects.

### Bleaching is an alternative approach for pigment removal but will compromise downstream applications

**Figure 8:**
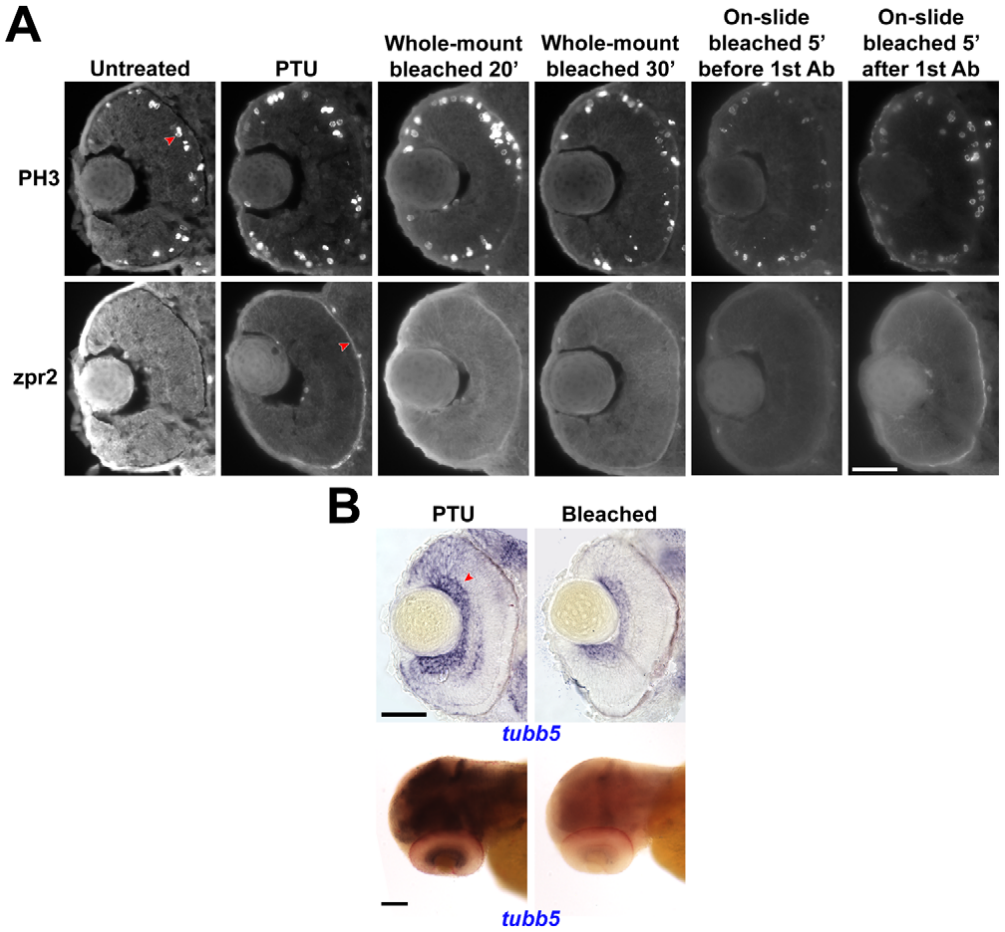
Performance of immunostaining and *in situ* hybridization after bleaching. (A) Immunostaining experiments were conducted with untreated, PTU-treated or bleached embryos at 52 hpf. Two first antibodies, anti-PH3 and zpr2 were used. The typical positive signal is indicated by a red arrowhead in each case. Scale bar  = 50 µm. (B) An *in situ* hybridization experiment with *tubb5* using PTU-treated or bleached embryos. These samples were obtained from a double *in situ* hybridization experiment with another gene stained in red; thus the samples, especially the whole-mount embryos, look reddish in general. Scale bar  = 50 & 100 µm for sectioned and whole-mount samples respectively. All images were acquired with the same parameters and were not altered during the figure composition to ensure comparability across conditions.

One alternative approach for removing pigment is to bleach fixed embryos. To determine the extent to which bleaching would affect the quality of immunostaining, a series of experiments were conducted on embryos treated with PTU or bleached with 3% H_2_O_2_/1% KOH [52] in different manners (Figure 8A). Two first antibodies, PH3 and zpr2, were used in this test. For PH3, the immunostaining signal in the sections of the bleached whole-mount embryos appeared comparable to the untreated or PTU-treated samples; however, the signal intensity was compromised if bleaching was conducted on sections. For zpr2, the signal background was higher in all samples that were bleached compared with the PTU-treated samples. Nonetheless, the signal that was obtained from bleaching the sections after incubation with the first antibody was the closest to the PTU-treated samples. Thus, different antigens will likely be affected by bleaching differently and the bleaching protocol would have to be optimized in each case. For *in situ* hybridization, a riboprobe against *tubulin, beta 5* (*tubb5*) was used (Figure 8B). The staining pattern of the whole-mount embryo was similar between the two groups; however, the signal of the bleached embryo was much weaker, and not all regions with positive signal in the PTU-treated embryos had discernible signal in the bleached samples. Together, these results suggest that while bleaching can potentially be an alternative for downstream investigations, PTU treatment for melanization inhibition remains the most effective approach.

## Discussion

This study has identified a specific effect of PTU treatment on eye size reduction in zebrafish larvae. The size reduction is not caused by a change in cell-type, proliferation or apoptotic status of the retinal cells, but is likely a result of tighter packing of these cells in the retina and a smaller lens. A chemical screen using different goitrogens and supplementing PTU-treated larvae with thyroid hormones have indicated the possibility of a reduction of TPO/ TPO-like activity rather than a general level of thyroid hormone to be the underlying cause of this PTU-induced effect on eye size reduction. The specific expression of *Tpo* in the developing mouse retina at P0 and a general expression in the lens and retina at E13.5 have provided strong corroborative evidence that a localized effect of TPO/ TPO-like activity in the eye region is potentially responsible for mediating an eye-specific outcome by PTU treatment.

This is not the first time that PTU treatment has been demonstrated to have an effect on growth and morphology. For example, a long-term 0.002% PTU treatment on five species of young tropical fish over eight weeks can depigment and shorten the body length (Length of treated fish vs. control, 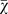 = 66.32%, *s* = 5.66%, species N = 5) [12]. In the current study, the body size has also been reduced by PTU treatment, but the present data have demonstrated that the eye size is specifically reduced to a greater extent, indicating a tissue-specificity of the treatment. Interestingly, Frieders also found that 0.0025% PTU treatment was too toxic for young fish used in his study [12]. His observations further suggest that the current standard treatment of zebrafish embryos with 1X PTU (0.003%) would cause unwanted physiological effects, as indicated by the current study and others as discussed above. One such effect is the inhibition of thyroid hormone synthesis. It is not surprising that altered thyroid activity would affect animal growth including the eye. In fact, a recent investigation of a rat model of thyroid hormone deficiency has demonstrated that a suppression of thyroid hormone by chemical inhibition and surgical thyroidectomy would reduce eye volume, optic nerve cross-sectional area and thickness of retinal layers [53]. However, the body weight gain is substantially lowered in this mouse model. Thus, it is unclear whether the morphological changes in the eye are a general effect of suppressing thyroid hormone production or there is a specific component to it. Further, it has been reported that a missense mutation in mouse *Tpo* causes hypothyroid dwarfism with a general body size reduction [54]. Presumably the eye size may be smaller, even though this was not investigated in that study. While these observations suggest that a normal thyroid activity contributes to a proper eye growth, the results of the current study indicate a possible additional layer of regulation by a TPO/ TPO-like activity that may specifically act in the proximity of the eye region.

How does the expression of a TPO/ TPO-like activity in retina and/or lens affect the general eye size? One possibility is through the modification of the extracellular matrix (ECM). TPO and similar peroxidases are expressed on cell surface [55]. It has been demonstrated that many of these enzymes create oxidative damage to ECM [56]; and a change in the strength and/or integrity of ECM components has been demonstrated to regulate eye size. For example, it has been observed in developing chick that by disrupting collagen formation in the vitreous body and the inner limiting membrane, the eye will be enlarged by 50% in 4 days [57]; and in a number of syndromic diseases that cause congenital high myopia, ECM proteins are mutated. The TPO/TPO-like activity in the developing eye may play a similar role in disrupting ECM by creating localized oxidative damage and this in turn allows for growth of eye tissue. Thus, the inhibition of TPO in the eye by PTU treatment will prohibit the eye from growing in the normal way and result in a smaller size. This change in eye size after PTU treatment may have implications in human refractive errors, in which the shape or size of the eye ball is affected by genetic, environmental and behavioral factors [58]. It is tempting to speculate that the mechanism as described above and the other signal transduction pathways that are altered by PTU-treatment may play a role in refractive errors, and that the regulation of the components in these pathways by pharmacological approaches may alleviate these eye conditions. However, early studies on PTU-treated [59] and *tyr* [60] larval eyes have demonstrated that they are emmetropic starting at 72 hpf and at 7 dpf respectively, arguing against the relationship between refractive errors and PTU treatment or pigmentation. Nonetheless, these studies have only investigated young larval eyes; for example, the oldest PTU-treated larvae that were used in the original study was 136 hpf and they were emmetropic [59]. It would be interesting to determine whether a long-term suboptimal dose of localized PTU treatment would ultimately induce refractive errors in older fish, or whether such application of PTU in older fish would alter the morphology of the eye ball.

PTU has been the most effective chemical inhibitor for melanization of zebrafish larvae. Our initial search for alterative inhibitors of tyrosinase was not successful. Among all the chemicals tested, only MBT is seemingly effective in inhibiting general melanization as reported [47]. Unfortunately, the treatment with MBT has specifically reduced the eye size to a much larger extent than the optimal PTU treatment. Indeed, MBT is also a TPO inhibitor [61] and can suppress thyroid hormone synthesis [62]; thus, the common inhibition of TPO by PTU and MBT potentially explains the similarities in the outcomes between these two chemical treatments. While bleaching is effective in removing pigment, it will compromise the efficiency of downstream investigations. Therefore, the findings from this and similar studies of PTU's effect on zebrafish development have highlighted an urgent need to search for novel chemical inhibitors of melanization with fewer side effects.

## Materials and Methods

### Fish maintenance and embryo collection

Wild-type (WT) zebrafish AB and AB/TL, *tyr^tk20/+^ (sandy/sdy)* [25] and *Tg(gfap:GFP)^mi2001^* [37] lines were used in this study. Maintenance, embryo collection, staging and incubation were performed according to standard procedures [17], [63]. The embryos used for an individual experiment were collected from the same parents and spawned within a 30-minute interval. All protocols were approved by the Purdue Animal Care and Use Committee.

### Mouse animals and housing

WT CD1 strain was used for producing embryos for *in situ* hybridization. All maintenance and procedures were performed as described [64]. All experiments were approved by the Animal Studies Committee at Washington University in St. Louis School of Medicine.

### Chemical Treatment

All chemicals used in this study were purchased from Sigma unless specified otherwise. They were dissolved in 1X E3 medium [18] (Table 1) and were used to treat embryos starting at 24 hpf. The control siblings were incubated in either 1X E3 or 1X E3 with the carrier of the particular chemical. The culture medium was changed daily. In some experiments, 1X or 0.3X PTU (Sigma and Alfa Aesar) was also included to inhibit melanization. The thyroid inhibitors used in this study were PTuracil, MM, DHP, RES, KClO4 and KSCN. The thyroid hormones used in this study were T4, T3 and TriAc. The tyrosinase inhibitors used include PTU, HPU, 4HBA, CPC, MQ and MBT. A literature review was conducted to select concentrations of these chemicals that would inhibit tyrosinase and exhibit least side effects. Then, a titration series of each chemical was tested to identify the highest possible concentration that did not induce major morphological abnormality, as judge by the deformation of the body and casualty at 5 dpf. The bioactivity of the thyroid hormones was further determined by qPCR as described below. The final concentrations of the thyroid hormones and inhibitors used in the study are as follows: 0.025% PTuracil, 4mM MM, 1 mM DHP, 0.5 mM RES, 0.2% KClO4, 10mM KSCN, 30 nM T4, 10 nM T3 and 0.1 µM TriAc. The study of the tyrosinase inhibitors was discussed in the results section.

### Morphological analysis

Fish larvae were positioned in 3% methylcellulose (Sigma). Images of the eye region and the whole body were acquired by a SPOT-RT3 colour slider camera (Diagnostic Instruments) mounted on an Olympus SZX16 stereomicroscope. Length and area measurements were conducted on lateral view images by i-Solution (IMT i-Solution). Body length was measured from the anterior tip of the snout to the posterior end of the caudal peduncle of the body. Eye length was measured from anterior to posterior of the eye along the longest axis. Eye and body areas were measured by tracing the boundary of the eye and the whole body excluding the fins respectively on the lateral images.

### Histological and immunohistochemical analyses

Histological analysis on 1-μm-thick plastic sections and immunohistochemistry on 10-μm-thick cryosections were conducted as described [65], [66]. To maximize comparability across different conditions, only sections with an optic nerve in the eyes on both sides were used. At least five embryos were collected and analyzed in each experimental group. Ganglion cells, photoreceptor cells, inner nuclear cells, and marginal zone cells were counted on both eyes by i-Solution (IMT i-Solution). Retinal and lens areas were also measured by tracing the boundary of the corresponding eye tissues or cell layers (IMT i-Solution). Whole-mount immunostaining of T4 was conducted using VECTASTAIN Elite Universal ABC Kit (Vector lab) [33]. The antibodies used in this study and their dilutions are as follows: mouse anti-zpr1 (1∶200, ZIRC), mouse anti-zpr2 (1∶750, ZIRC), mouse anti-zpr3 (1∶200, ZIRC), mouse anti-Islet1 (1∶50, Developmental Studies Hybridoma Bank), mouse anti-acetylated α-tubulin (1∶1000, Sigma), rabbit anti-phospho-Histone H3 (PH3) (1∶500, Millipore), rabbit anti-active caspase3 (1∶500, BD Biosciences), rabbit anti-T4 (1∶4000; MP Biomedicals), Alexa Fluor 488/555 goat anti-rabbit/mouse IgG (1∶1000, Invitrogen). The resulting sections were imaged by a SPOT-RT3 camera mounted on an Olympus BX51 fluorescence compound microscope while the whole-mount samples were imaged as described in the morphological analysis section. The images were subsequently merged using Adobe Photoshop CS3 (Adobe).

### *In situ* hybridization

*In situ* hybridization of *Tpo* was conducted on 20-μm-thick cryosections of mouse embryos at embryonic day 13.5 (E13.5) and postnatal day 0 (P0) as described [64]. A plasmid containing the mouse *Tpo* gene (GenBank Accession Number: BC099421) was purchased from Thermo Scientific. Linearized DNA template for *in vitro* transcription of the riboprobe was generated by PCR, using M13 primers with an additional T7 site on the forward primer and T3 on the reverse primer. *In situ* hybridization of *tubb5* in zebrafish was conducted as described [67].

### Bleaching

To investigate the performance of bleaching for downstream applications including immunostaining and *in situ* hybridization, embryos were bleached in 3% H_2_O_2_/1% KOH [52] in different manners. This includes bleaching whole-mount embryos for 20 or 30 minutes after fixation, and bleaching cryosectioned samples collected on a slide for five minutes before or after incubation with the first antibody. The samples were then analyzed by regular protocols for immunostaining and *in situ* hybridization after being washed extensively.

### Quantitative PCR (qPCR)

Larval collection, total RNAs purification and quality checks, and reverse transcription were conducted as described [68]. qPCR was conducted using SYBR Green PCR Master Mix (Applied Biosystems) on an Applied Biosystems 7300 Real-Time PCR System according to the manufacturer's instructions. Primers were designed using Primer Express Version 3.0 (Applied Biosystems) with standard parameters. The following primer pairs were used in this study to amplify *D2*, *TRα* and *β-actin (β-act):* D2-FP: 5′-CATCAGCGGTAAGACCCACAA-3′, D2-RP: 5′-AAGACCGGCAGCTGGCTTATA-3′, TRα-FP: 5′-CTATGAACAGCACATCCGACAAG-3′, TRα-RP: 5′-CACACCACACACGGCTCATC-3′, β-act-FP: 5′-TGCTGTTTTCCCCTCCATTG-3′.

β-act-RP: 5′-GTCCCATGCCAACCATCACT-3′.

### pH and osmolarity measurement

pH and osmolarity of chemicals were measured by an Orion 2 Star pH meter (Orion) and an Osmette A Automatic Osmometer (Precision Systems) respectively.

### Statistical analysis and data visualization

All standard descriptive statistics and data analyses were performed in the R statistical environment (http://www.r-project.org) version 2.11.1. The analysis of the collected data was conducted by Wilcoxon rank sum test for two groups and ANOVA for three or more groups. Multiple hypothesis testing correction for Wilcoxon rank sum tests was conducted by the Holm's method. *Post hoc* comparisons for ANOVA were conducted using Bonferroni-corrected *t-*test. Linear mixed-effects model was built to delineate the specific effect on eye growth by chemical treatment, with the experiments conducted on different days modeled as a random effect [69]. qPCR data were analyzed by the ΔΔCt method [70] implemented in the *ddCt* package in Bioconductor (http://www.bioconductor.org/). The qPCR results were reported in fold change (2̂ddCt) and the corresponding range in 2̂(ddCt ± SE). Standard error propagation was used to combine measurement errors of the variables. An alpha level of 0.05 was used for all statistical tests. Heatmaps were generated using Multiexperiment Viewer (MeV) (http://www.tm4.org).
